# PharmRL: Pharmacophore elucidation with Deep Geometric Reinforcement Learning

**DOI:** 10.21203/rs.3.rs-5033986/v1

**Published:** 2024-09-24

**Authors:** Rishal Aggarwal, David R. Koes

**Affiliations:** 1 Joint PhD Program in Computational Biology, Carnegie Mellon University-University of Pittsburgh, Pittsburgh, Pennsylvania.; 2 Computational & Systems Biology, University of Pittsburgh, Pittsburgh, Pennsylvania.

**Keywords:** Pharmacophores, Virtual Screening, Protein-Ligand interactions, Machine Learning

## Abstract

Molecular interactions between proteins and their ligands are important for drug design. A pharmacophore consists of favorable molecular interactions in a protein binding site and can be utilized for virtual screening. Pharmacophores are easiest to identify from co-crystal structures of a bound protein-ligand complex. In this work, however, we develop a deep learning method that can identify pharmacophores in the absence of a ligand. Specifically, we train a CNN model to identify potential favorable interactions in the the binding site, and develop a deep geometric Q-learning algorithm that attempts to select an optimal subset of these interaction points to form a pharmacophore. With this algorithm, we show better prospective virtual screening performance, in terms of F1 scores, on the DUD-E dataset than random selection of ligand identified features from co-crystal structures. We also conduct experiments on the LIT-PCBA dataset and show that it provides efficient solutions for identifying active molecules. Finally, we test our method by screening the COVID moonshot dataset and show that it would be effective in identifying prospective lead molecules even in the absence of fragment screening experiments. Alongside, we provide a Google Colab notebook for ease of use of the developed method.

## 1 Introduction

**Fig. 1:**
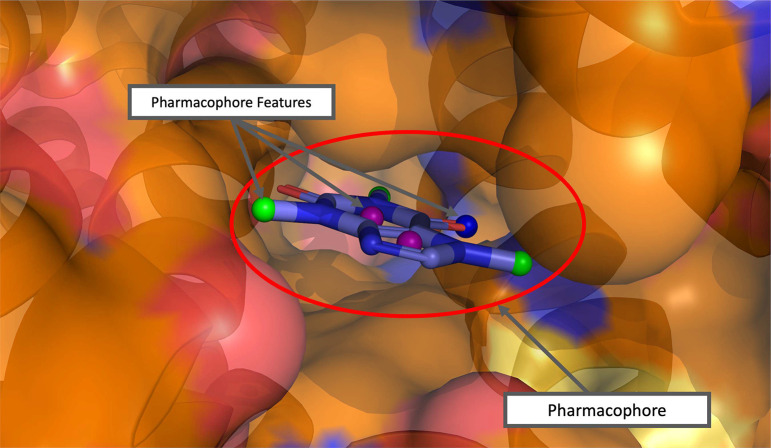
Pharmacophore model with several pharmacophore features that matches the caffeine molecule (caffeine molecule included for illustration). The colors of the feature points are as follows: Aromatic - Purple, Hydrogen Acceptor - Blue, Hydrophobic - Green.

An essential part of computer-aided drug design is elucidating important molecular interactions between proteins and their ligands. One way to describe these molecular interactions is by depicting a 3D arrangement of protein-ligand interaction features known as a pharmacophore. A pharmacophore is a set of interaction features, also known as pharmacophore features, that describe the favorable interactions between a protein binding site and a ligand. It can be used for screening large libraries through efficient pattern matching algorithms implemented by open source softwares such as Pharmit[1, 2]. An example pharmacophore is shown for the caffeine molecule in Figure 1.

Developing a useful pharmacophore typically requires a co-crystal structure of a protein and its cognate ligand. This is because bound structures provide enriched features that can be considered ground truth favorable interactions for protein-ligand binding. This requirement can be a significant obstacle in many real-world drug discovery projects, where bound co-crystal structures are often unavailable.

Traditional methods for generating pharmacophore features in the absence of a ligand typically involve introducing molecular fragment probes into the binding site to identify areas with high affinity [3–5]. Another strategy involves molecular dynamics simulations of the target protein in a simulation environment containing probe molecules with varying chemical properties. This helps pinpoint regions where these probes occur frequently[6]. Subsequently, experts manually select and combine a subset of these interaction features to construct a concise pharmacophore[7, 8]. FRESCO[9] follows a novel approach that avoids filtering features. They use the fit of molecules on distributions of pharmacophore feature distances to rank molecules.

Once interaction features are identified, they need to be ranked and grouped together to form a pharmacophore. Several methods exist for ranking interaction features at binding sites. These methods involve the calculation of interaction energies at feature points[10], while others focus on identifying key pocket atoms for binding[11] and prioritize interaction features in close proximity to these atoms. Recently, a method known as Apo2ph4 [12] was developed for automating the selection process of a subset of pharmacophore features. Apo2ph4 evaluates each feature point by considering both the proximity of other similar features and the interaction energies associated with that point. The resulting pharmacophore is then composed of features whose scores exceed a predetermined threshold. Finally, in certain limited cases, homology models may also be used to elucidate pharmacophores[13, 14]. However, these approaches have notable limitations.

Firstly, the pharmacophore features obtained through these methods are influenced by the biases inherent to the docking and simulation protocols employed. Secondly, the final step of constructing the pharmacophore heavily relies on human insight. Furthermore, methods that try to filter out pharmacophore features evaluate each feature in isolation rather than considering its contribution to a fully-formed pharmacophore. These factors collectively underscore the need for more experimental, data-driven approaches to generate pharmacophore features. Additionally, there is a need for the development of automated tools for pharmacophore modeling that can still benefit from expert guidance.

To address this requirement, we developed a CNN model and a deep geometric Q-learning algorithm to identify interactions features and elucidate pharmacophores. The method is demonstrated to strong performance in retrospective virtual screening experiments across several datasets such as Dataset of Useful Decoys - Enhanced (DUD-E)[15], LIT-PCBA[16], and COVID Moonshot[17].

## 2 Method

**Fig. 2:**
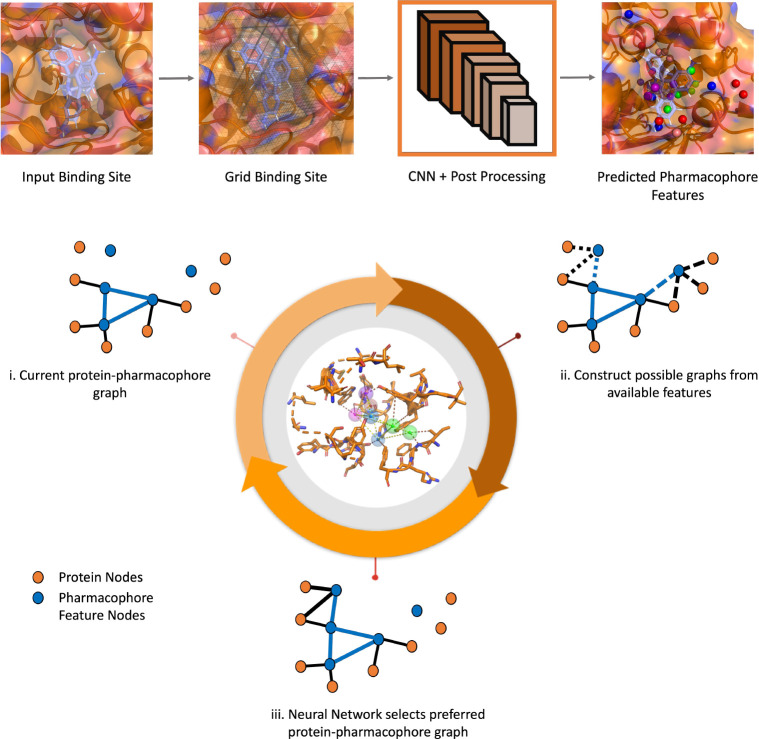
Pharmacophore prediction pipeline. CNN is used to predict pharmacophore features from gridded binding site (Top). Protein - pharmacophore graph is built by sequentially adding feature and protein nodes to it using RL framework (Bottom).

In this method, we trained a convolutional neural network (CNN) model to identify favorable points of interactions (pharmacophore features) on the binding site and developed a deep geometric Q-learning algorithm that attempts to select an optimal subset of these interaction points to form a pharmacophore. The CNN model is trained on pharmacophore features derived from protein-ligand co-crystal structures and is iteratively fine tuned with adversarial examples to ensure predicted points of interaction are physically plausible and close to relevant functional groups on the protein. The reinforcement learning algorithm employs an SE(3)-equivariant neural network[18] as the Q-value function. This network progressively constructs a protein-pharmacophore graph. It does so by either choosing to incorporate an available pharmacophore feature into the graph or determining that the current graph is already optimal. The pipeline for the method is shown in Figure 2. Importantly, this framework still has the ability to accommodate expert guidance in selecting and adding features while automating a significant portion of the traditional pharmacophore elucidation process.

### 2.1 Pharmacophore Definition

A pharmacophore is defined as a set of points $\left\{V_{f}\right\}$ that propose positions of interactions between the give protein binding site and a potential ligand. More specifically each point in a pharmacophore has a 3D coordinate $X_{f}\in R^{3}$ and feature class $Z_{f}$. The feature class is defined to be any of the following: {Hydrogen Acceptor, Hydrogen Donor, Hydrophobic, Aromatic, Negative Ion and Positive Ion}. Pharmacophore search software such as Pharmit[1] can be used to retrieve molecules that can satisfy the feature and position constraints specified by a given pharmacophore. In this work, pharmacophores are developed in two major steps. First, potential points of interactions on a binding site are identified using a convolution neural network (CNN). A subset of these identified points are then selected with a reinforcement learning model to form a pharmacophore. More details follow in subsequent sections.

### 2.2 CNN training

The CNN model is trained to predict whether a given point on the binding site is a plausible point of interaction. Specifically, the CNN predicts if any of the six feature classes are present at the given point. It is trained in a multilabel classification manner so that it can predict the presence of multiple classes at the evaluated point (e.g. Hydrogen Donor and Hydrogen Acceptor for a hydroxyl). The CNN takes as input, a voxelized representation of the protein structure located in a cubic volume of edge 9.5 Å, at a resolution of 0.5 Å, centered at the point. The libmolgrid[19] python library with its default atom types is used for voxelizing the protein structure. The CNN model architecture and further training details are provided in Supplementary section 1.2. The model is initially trained on pharmacophore features extracted from the PDBBind V. 2019 dataset[20]. These features are considered enriched as they represent “ground truth” interactions between the receptor and its cognate ligand. The Pharmit search software is used to identify these features. Pharmit utilizes SMART strings[21] and distance-based heuristics to pinpoint the interacting feature positions on the ligands. More details on our dataset are provided in Supplementary section 1.1.

To enhance the robustness of pharmacophore feature predictions, the CNN undergoes retraining with adversarial samples. Adversarial samples are generated through a two-step process. Firstly, the protein binding sites are discretized at a resolution of 0.5 Å, and the CNN is evaluated at each grid point. Predictions that are too close to protein atoms are labeled as negative. Additionally, predictions where complementary functional groups of interest on the protein are too distant are collected as adversarial samples. For instance, Hydrogen Acceptor predictions beyond 4 Å from any hydrogen donor functional group on the protein are considered negatives. Thresholds for pharmacophore features and their complementary functional groups are outlined in Supplementary section 1.4.

### 2.3 From CNN predictions to pharmacophore features

Pharmacophore features are individual interaction points found in the binding site. One key assumption is that these features should be in proximity to complementary interaction feature groups on the protein. These features are inferred through a multistep process.

**Fig. 3:**
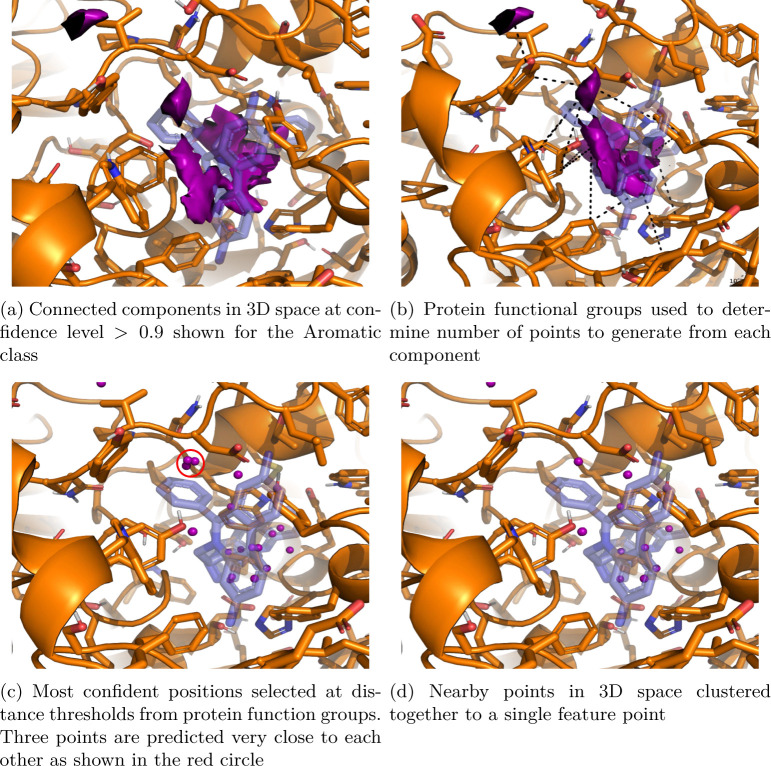
Steps followed to obtain pharmacophore feature points from a CNN predictions on a binding site

The pharmacophore generation process can be viewed in Figure 3. As before, the binding site is first gridded at a resolution of 0.5 Å and the CNN is evaluated at each grid point. This results in a dense grid of feature confidences. Once this is done we need to determine the number of feature points that have to be extracted from each connected component. We use the complimentary functional groups on the protein that are close to the connected components (Figure 3b) to determine the number of feature points by taking the top predicted point (Figure 3c) within a distance threshold (refer to Table 2 for the thresholds) to it.

Finally, feature points are refined by grouping predictions that are near each other through agglomerative clustering. A distance threshold of 1.5 Å is used as criteria for merging clusters and the centroid of each cluster is taken as the predicted pharmacophore feature (Figure 3d).

### 2.4 Formation of pharmacophores from features

A subset of the candidate pharmacophore features are selected to form a full pharmacophore. This process is modelled as a reinforcement learning problem. Specifically, the method is a deep Q-learning framework that utilises a SE(3)-equivariant neural network[18] to model the Q value function. The RL algorithm is trained on the Dataset of Useful Decoys - Enhanced (DUD-E) dataset as it provides an extensive set of actives and decoys for each protein-ligand system in its dataset.

### 2.5 Why Reinforcement Learning

Modeling pharmacophores presents a substantial challenge because it involves selecting a concise set of features suitable for virtual screening. Pharmacophores are built by combining specific features, and this combination greatly influences their performance. Notably, adding or removing a single feature can significantly impact the overall performance, making it challenging to assess the individual importance of each feature in isolation. This complexity poses a hurdle for traditional supervised learning approaches, such as the CNN.

However, reinforcement learning (RL) offers a different perspective. RL has the potential to consider the long-term consequences of adding a single feature to a pharmacophore. Consequently, a RL algorithm can sequentially incorporate features into a pharmacophore model while considering the overall value of the fully formed pharmacophore, rather than just the immediate value of each individual feature added along the way.

### 2.6 Pharmacophore selection as a Markov Decision Process (MDP)

The generation of the pharmacophore follows an iterative process via the construction of a heterogeneous 3D graph. The graph contains “pharmacophore” nodes ($\left\{V_{f}\right\}$) representing pharmacophore features and “protein nodes” ($\left\{V_{p}\right\}$) that contain the protein atoms in proximity of the bespoke pharmacophore features. Each iteration involves adding pharmacophore feature nodes and their associated protein atoms to the graph. The structure of the graph in the next step depends entirely on its current state, making the process akin to a Markov decision process (MDP).

In the context of reinforcement learning, a Markov Decision Process (MDP) is defined with a set of states s∈S that provide information of the environment, actions a∈A that help in moving from the current state to the next state, and a reward function R(s,a)→R that provides a reward value for state-action pair.

Here, at a given time-point (t) in the iterative process, we define a state $\left(s_{t}\right)$ as a heterogeneous protein-pharmacophore graph denoted as $G_{t}\left(V_{f},V_{p},E_{f,f},E_{f,p}\right)$, consisting of pharmacophore feature nodes $\left(V_{f}\right)$, protein nodes $\left(V_{p}\right)$, and edges ($E_{f,f}$ and $E_{f,p}$) connecting feature nodes to features and protein nodes. The edges are formed based on predefined distance thresholds $\delta_{f,f}$ and $\delta_{f,p}$.

This definition leads to a set of possible states $\left\{s_{t+1}\right\}$ that can be reached at time-point t+1 by considering the addition of a feature not present in the current graph but within a distance of $\delta_{f,f}$ from any feature node in the graph. This results in a set of proposed graphs denoted as $\left\{G_{t+1}\right\}$. The current graph $G_{t}$ is also added to this set, forming a superset $\left\{s_{t+1}\right\}=\left\{\left\{G_{t+1}\right\},G_{t}\right\}$. The action $\left(a_{t}\right)$ then involves selecting one of the graphs from this proposal set as the next state. If the current graph is selected, the process terminates.

**Fig. 4:**
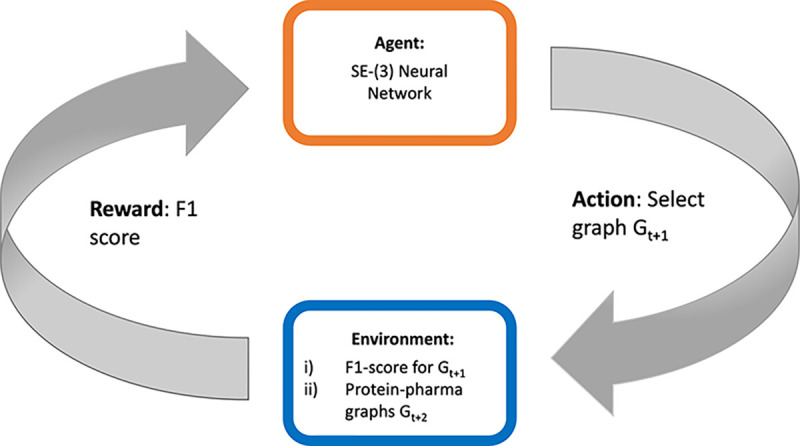
MDP process used for iterative construction of the protein-pharmacophore graph. At each time-point t, the action is to chose the next graph ($G_{t+1}$). The environment takes this and provides a F1-score for that pharmacophore, along with possible graphs to choose from ($\left\{G_{t+2}\right\}$) for the next iteration.

The reward for each step $r_{t}=R\left(s_{t},a_{t}\right)=R\left(G_{t+1}\right)$ is calculated based on the F1 score obtained by running the pharmacophore, obtained as a combination of the features nodes in the graph $G_{t+1}$, on a dataset containing actives and decoys. Pharmit, the tool used, requires a pharmacophore with at least 3 nodes to screen molecules. Therefore, until the current graph has at least 3 nodes, we assign a reward of 0 and do not include the current graph in the proposal set. A schematic representation of the MDP process at time-point t is given at Figure 4.

### 2.7 Deep Q-Learning

The objective of reinforcement learning is to learn a policy $\pi^{*}:S\to A$ that maximizes the cumulative (discounted) reward you obtain from a MDP. In Q-learning, the function Q(s,a) is trained to predict future rewards given an action on a state. In this context, for a policy π the Q-value of a state action pair is given by:

(1) $$Q^{\pi }\left(s_{t},a_{t}\right)=Q^{\pi }\left(G_{t},a_{t}\right)=Q^{\pi }\left(G_{t+1}\right)=E\left[\sum_{i=t}^{T}\;\;\gamma^{i-t}\;*\;r_{i}\right]$$

Where γ is a predetermined reward discount factor. The discount factor implicitly weighs the importance of the immediate reward with respect to the cumulative reward. The optimal policy defined at a state then is $\pi^{*}(s)={argmax}_{a}Q^{\pi^{*}}(s,a)$. For this problem this equation translates to:

(2) $$\pi^{*}\left(G_{t}\right)={argmax}_{G_{t+1}}Q^{\pi^{*}}\left(G_{t+1}\right)$$

**Fig. 5:**
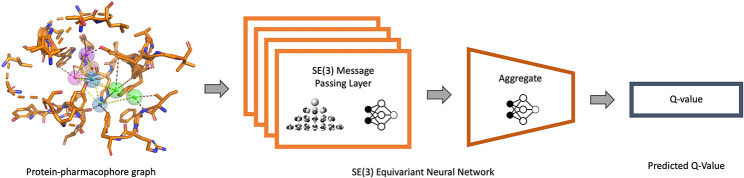
The SE(3)-equivariant neural network takes a protein-pharmacophore graph as input and predicts the Q-value

A SE(3)-equivariant neural network is used to parameterize the Q function (Figure 5). The neural network is trained to minimize the objective $l(\theta )=E\left[y_{t}-Q\left(G_{t};\theta \right)\right]$ where θ is the parameter set of the neural network and $y_{t}$ is given by:

(3) $$y_{t}=r_{t}+\gamma \;*\;{max}_{G_{t+2}}\;Q\left(G_{t+2};\theta \right)$$

### 2.8 Training Details

The DUD-E dataset is split into training and test sets, with the test set being the diverse subset of the DUD-E dataset. This subset contains 8 proteins that are representative of all the proteins in the dataset. The neural network operates on a protein-pharmacophore graph as input. Protein nodes are represented by atom types, while pharmacophore nodes take as features the output of the final hidden layer of the CNN. The final hidden layer of the CNN can be interpreted as an embedding of the local environment around the feature point. It provides a latent vector of size 32. Initially, the model is trained using pharmacophore features extracted from protein-ligand co-crystal structure and is then fine-tuned using pharmacophore features obtained from CNN predictions. Since the ligand features are obtained from crystal structures, they also have directional information about aromatic/hydrogen bonding interations between the ligand and the protein which are used while evaluating generated pharmacophores. A hyperparameter sweep was also conducted while training on pharmacophore features extracted from the cognate ligand. For a detailed list of hyperparameters, refer to Supplementary section 3.4. The model that provides the best mean F1-score on ligand extracted features is used to train an ensemble of 5 models on CNN-predicted features.

The training algorithm goes through the protein-ligand systems in the training set, generating training samples through episodes simulated using an ϵ-greedy policy. ϵ-greedy balances exploration and exploitation by setting a probability ϵ by which a random action is taken as compared to taking the action decided by the neural network. While training the epsilon decays exponentially according to the equation $\epsilon_{t}=\epsilon_{T}+\left(\epsilon_{o}-\epsilon_{T}\right)\;*\;e^{\left(-t/\alpha \right)}$, where $\epsilon_{o}$ and $\epsilon_{T}$ are initial and final epsilons, and α is a predetermined decay rate parameter. Using this, the initial iterations of RL training is focused on exploring as many graphs as possible. Later iterations are focused on optimizing the learnt policy based on the graphs sampled by the neural network as the neural network has better graph proposals. While training on ligand based features, an $\epsilon_{o}$ value close to 0.9 is used, but when fine-tuning on CNN features $\epsilon_{o}=0.5$ is used as lesser amount of exploration is required at this stage. Additional training specifics can be found in Supplementary section 3.3. The metrics we use to evaluate the algorithms performance can be viewed in Supplementary section 4.

## 3 Results

We show that this algorithm has the potential to provide performant solutions in virtual screening experiments on the Dataset of Useful Decoys - Enhanced (DUD-E)[15] and LIT-PCBA[16] datasets. We also test the method on screening the COVID Moonshot dataset[17] and show that it would provide pharmacophores with the ability to identify binding molecules even in the absence of fragment screening experiments.

### 3.1 CNN models successfully classify pharmacophore feature points

The CNN model is initially trained using features extracted from co-crystal structures. It’s worth emphasizing that every data point in this dataset is associated with at least one of the classes, and in such cases, the model accurately predicts the corresponding classes with high precision. The ROC-AUC for each class surpasses 0.95, and detailed class-specific ROC-AUC scores can be found in Supplementary Section 1.3. Subsequently, the CNN undergoes retraining using adversarial examples to enhance the robustness of its predictions during inference. Importantly, this retraining has a negligible impact on the model’s classification performance.

Furthermore, certain false positives are accounted for by ensuring that generated features are at appropriate distance from the relevant function groups on the protein. The feature prediction algorithm provides an average of 136 features per a binding site in the DUD-E dataset. An example of what this looks like is shown in Supplementary Figure 2.

### 3.2 RL models provide at least one good solution on the DUD-E diverse subset

The diverse subset of the DUD-E dataset is used to test the RL algorithm. This subset, provided by the developers of the DUD-E dataset, represents all the protein classes present in the dataset. For each system in the DUD-E dataset, all possible combinations of pharmacophore features from the cognate ligand are enumerated. Since the best possible F1 score differs from system to system, the F1 score normalized by the maximum possible F1 achievable from the ligand features is reported. We notice that the best F1 score across all systems are from pharmacophores that are either of size 3,4, or 5; therefore, the mean of all possible pharmacophores of max size 5 are considered as a random selection baseline.

To evaluate the performance of a supervised learning approach for pharmacophore generation that ranks pharmacophore features individually, combinations of the top-3, top-4 and top-5 CNN ranked features are used as pharmacophores. Each of these pharmacophores, except one (F1 = 0.028), yield an F1 score of 0, indicating that a supervised approach trained on individual features is not sufficient for this problem and a RL approach is more ideal.

From this point onward, we will refer to the RL models trained on CNN features as PharmRL_CNN and the model trained on ligand features as PharmRL_Ligand. During training on the DUDE set, we observed that the models tend to converge on generating pharmacophores with only 3 features. This is likely due to the large number of actives in the DUDE dataset, which drives the model to prioritize enhancing recall performance. Because these pharmacophores lack selectivity, we also evaluate the models when they are required to generate pharmacophores with at least 4 features.

**Fig. 6:**
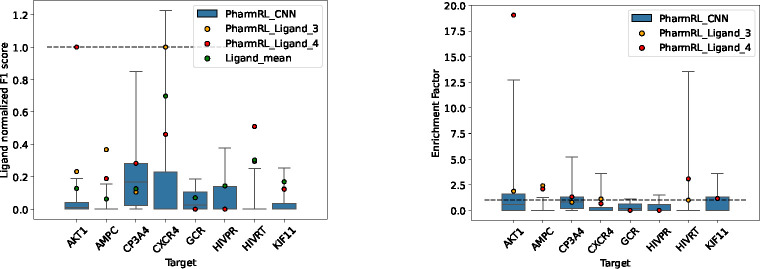
F1-scores divided by the max F1 score attainable from ligand features for RL models trained and tested on ligand derived features (PharmRL_Ligand) and all CNN features (PharmRL_CNN)

Figure 6 showcases the results of the pharmacophores generated from the application of our RL models on the 8 test systems. Each box plot is a culmination of 10 pharmacophores (minimum 3 and 4 features) from our CNN based models. The whiskers of the box plot are set to maximum and minimum value of the set. The performance of PharmRL_Ligand is also shown with a minimum of 3 (PharmRL_Ligand_3) and 4 features (PharmRL_Ligand_4). Ligand mean is the F1 score obtained from the aforementioned random selection baseline. All the F1 scores are normalized by the max possible F1 score attainable from the ligand features. We also report other metrics for these models in Supplementary Table 5.

From Figure 6, it is clear that for each system the models have generated a pharmacophore that does better than the average of random selection. The method consistently generate at least one pharmacophore that achieves an enrichment factor greater than 1, indicating that it performs better at identifying active compounds than random selection from the dataset. The model trained and tested on ligand features finds the best achievable solution in 2/8 systems indicating the model is capable of finding the right pharmacophore from ligand features on certain systems. For 5/8 systems a model that uses the CNN predicted features are able to provide a pharmacophore that has better performance than the model trained on ligand-only features. Notably, for one of those systems (CXCR4), the solutions provide a F1 score that is higher than that of the max F1-score achievable from the ligand-only features. This is empirical evidence that the CNN is able to predict pharmacophore features that are relevant in the context of the given binding site and could be used for molecular screening. The RL algorithm, however, is necessary to assemble pharmacophores in an automated way.

### 3.3 RL models identify effective pharmacophores for COVID Moonshot

To test the screening capability of the RL models on the SARS-CoV2 Mpro protein we used a dataset of 23 publicly released non-covalently bound protein-fragment structures[22]. The identified pharmacophore features from the 23 complexes are clustered together in 3D in the same manner as for the CNN features. We also generate CNN based pharmacophore features in the binding sites using one of the structures. We screen against the COVID Moonshot dataset and label active molecules as those molecules that have an IC_50_ value < 5*μ*M. This evaluation was carried out in two retrospective phases. The “hit-to-lead” phase encompassed 979 molecules deposited before September 1st, 2020, of which 6% are considered actives[9]. The complete dataset, which represents the most up-to-date information, comprises 2,062 molecules, of which 38% are considered actives.

**Fig. 7:**
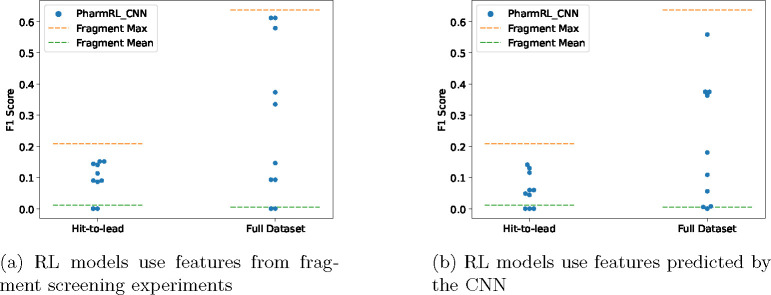
Performance of RL models on Covid screening experiments

Since it is computationally intractable to enumerate all possible pharmacophores from the fragment features, we sample 10,000 pharmacophores of sizes of 3,4 or 5 and report the max and mean F1 score obtained (Fragment_Max and Fragment_Mean). We compare this to the performance of our RL models on fragment features (Figure 7a) and CNN predicted features (Figure 7b). It’s important to emphasize here that the F1 scores presented in this experiment are the actual F1 scores and not max-normalized F1 scores. We also report other metrics for these models in Supplementary Table 7.

In both cases we can see that the RL models find pharmacophores that are close to the optimal F1-scores. This is exciting as it is an indication that the RL models can be used in tandem with pharmacophore features with “ground truth interactions” derived from experimentally determined fragment structure complexes. Furthermore, in most cases the RL models perform significantly better than random for feature selection. Perhaps more exciting is that the CNN + RL framework was able to identify find good pharmacophores even in the absence of any fragment data. We provide example pharmacophores and further analysis in Supplementary Section 5.

### 3.4 PharmRL has comparative performance to baselines on the LIT-PCBA dataset

Finally, the performance of PharmRL is evaluated and compared to Apo2ph4[12] on the LIT-PCBA dataset. To perform this comparison, we directly use the pharmacophores provided by the Apo2ph4 authors. Since their screening procedure involves proprietary software, we decided to create an open source benchmark using their pharmacophores. Therefore, we screen their pharmacophores on the LIT-PCBA dataset using pharmit with receptor exclusion turned on. The same parameters are used to screen our pharmacophores.

**Fig. 8:**
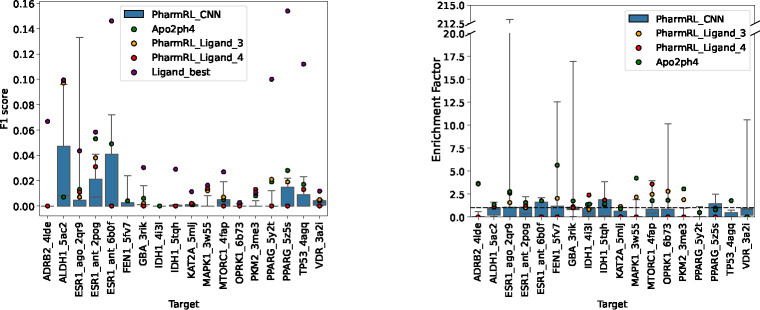
Performance of pharmacophore models on LIT-PCBA targets

Figure 8 provides the results of the pharmacophore models on the LIT-PCBA systems. The legend is consistent with the one defined beforehand in Section 3.2. Note that we report actual F1 scores rather than ligand-normalized F1 scores. However, we do provide the best achievable F1 score based on ligand features, labeled as Ligand_best. Additional metrics for pharmacophore performance are reported in Supplementary Table 8.

From the figure, it is evident that PharmRL_CNN provides at least one pharmacophore that achieves a better F1 score and enrichment factor than Apo2ph4 on 12 out of 18 systems. On one of the systems (ESR1_ago), a pharmacophore even outperforms the best attainable performance based on ligand features, further indicating that the CNN provides relevant features for the RL models to screen. The method also yields pharmacophores with an enrichment factor greater than 1 for all systems except ADRB2, demonstrating that some of the pharmacophores possess significant screening strength.

## 4 Discussion

In this work, we provide a framework to elucidate pharmacophores on a given binding pocket using only the protein structure. This is particularly important when co-crystal structures with cognate ligands do not exist. To accomplish this, we employ a CNN model to predict the potential locations of pharmacophore features within the binding site. Subsequently, these predictions are fed into an RL algorithm that utilizes a rotational equivariant neural network to generate pharmacophores that are subsets of these features.

The CNN model is trained using features extracted from co-crystal structures found in the PDBbind V. 2019 database. Since the prediction of pharmacophore features should rely solely on the local context surrounding a specific point, we input only the minimal local information into the CNN. The CNN demonstrates high accuracy in identifying the correct features at positions provided by the structures in the training set (Supplementary Table 1, Supplementary section 1.3). However, it needs to be retrained with adversarial samples to ensure that its predictions during the inference stage are physically plausible and relevant.

As demonstrated above (Figures 6, 7 and 8), the pharmacophores generated using these features and our RL model exhibit strong retrospective virtual screening performance, indicating the model’s ability to provide the correct features at relevant positions within the binding site. Additionally, in certain cases, as evidenced by the higher F1 score (system CXCR4 Figure 6, Esr1_ago Figure 8), the CNN can offer features that are more effective than those from the cognate ligand structures.

Reinforcement learning is employed to create pharmacophores from features, as a supervised approach on individual features proves inadequate for this task. Another challenge arises from the limited availability of virtual screening data to train models for distinguishing effective and ineffective pharmacophores. Additionally, the dataset may inherently contain biases regarding what constitutes a proficient pharmacophore within a binding pocket. For example, if a system predominantly consists of a congeneric series as its active molecules, relying solely on the F1-score can lead to skewed results, where highly rewarded pharmacophores may exclusively match molecules from that specific series. Furthermore, in the context of protein-ligand binding, a binding site has the potential to bind to multiple diverse types of ligands. Hence, there’s no singular correct pharmacophore for a particular binding site. Consequently, this leads to substantial variability in the optimal policy for this task and thus a large variance in the policy learnt by the RL models.

It is important to note that we don’t expect great performance all the time even from a perfect model - the model may generate pharmacophores that match active molecules whose chemotypes are not present in a retrospective screening. Therefore, five RL models are trained to generate pharmacophores, with the aim of mitigating the inherent variability in the problem and accounting for potential biases in the dataset. However, the training is also constrained by the limited availability data with the DUD-E dataset containing only 102 systems. As previously demonstrated, the RL models consistently produce at least one effective pharmacophore for most test system in the dataset (refer to Figures 6,7). It’s important to emphasize that while these models are compared against randomly selected ligand features, the ligand features are “ground truth” interaction points, making the pharmacophores generated from them inherently enriched. Finally, we also compare to an established baseline (Apo2ph4) on the LIT-PCBA dataset and show the models exhibiting comparable performance to the baseline.

Our method offers notable advantages as it is completely open source and it has the capacity for human intervention. The sequential nature of the graph-building framework grants users the opportunity to decide which features should be included or excluded in the generated pharmacophore. Furthermore, users can control the size of the generated pharmacophore, enabling the addition or removal of nodes as required. Users could also incorporate features obtained from other methods such as fragment experiments and ensure that they are present in the generated pharmacophore. These user-friendly tools are implemented and easily accessible in a google colaboratory notebook (link) and the full open source code for training and inference is available at https://github.com/RishalAggarwal/Pharmrl.
